# Systemic Lupus Erythematosus with and without Anti-dsDNA Antibodies: Analysis from a Large Monocentric Cohort

**DOI:** 10.1155/2015/328078

**Published:** 2015-05-06

**Authors:** Conti Fabrizio, Ceccarelli Fulvia, Perricone Carlo, Massaro Laura, Marocchi Elisa, Miranda Francesca, Spinelli Francesca Romana, Truglia Simona, Alessandri Cristiano, Valesini Guido

**Affiliations:** Lupus Clinic, Reumatologia, Dipartimento di Medicina Interna e Specialità Mediche, Sapienza Università di Roma, Roma 00161, Italy

## Abstract

*Objectives*. The anti-dsDNA antibodies are a marker for Systemic Lupus Erythematosus (SLE) and 70–98% of patients test positive. We evaluated the demographic, clinical, laboratory, and therapeutical features of a monocentric SLE cohort according to the anti-dsDNA status.* Methods*. We identified three groups: anti-dsDNA + (persistent positivity); anti-dsDNA ± (initial positivity and subsequent negativity during disease course); anti-dsDNA − (persistent negativity). Disease activity was assessed by the European Consensus Lupus Activity Measurement (ECLAM).* Results*. We evaluated 393 patients (anti-dsDNA +: 62.3%; anti-dsDNA ±: 13.3%; anti-dsDNA −: 24.4%). The renal involvement was significantly more frequent in anti-dsDNA + (30.2%), compared with anti-dsDNA ± and anti-dsDNA − (21.1% and 18.7%, resp.; *P* = 0.001). Serositis resulted significantly more frequent in anti-dsDNA − (82.3%) compared to anti-dsDNA + and anti-dsDNA ± (20.8% and 13.4%, resp.; *P* < 0.0001). The reduction of C4 serum levels was identified significantly more frequently in anti-dsDNA + and anti-dsDNA ± (40.0% and 44.2%, resp.) compared with anti-dsDNA − (21.8%, *P* = 0.005). We did not identify significant differences in the mean ECLAM values before and after modification of anti-dsDNA status (*P* = 0.7).* Conclusion*. Anti-dsDNA status influences the clinical and immunological features of SLE patients. Nonetheless, it does not appear to affect disease activity.

## 1. Introduction

Systemic Lupus Erythematosus (SLE) is an autoimmune disease, characterized by the production of a wide range of autoantibodies, resulting from polyclonal B cells activation, impaired apoptotic pathways, or idiotypic network dysregulation [1–5].

The anti-double stranded DNA antibodies (anti-dsDNA) are considered a specific marker for SLE [6]. Due to the high frequency (ranging from 70% to 98%), sensitivity, and specificity (57.3% and 97.4%, resp.), the presence of these autoantibodies could be virtually diagnostic for SLE [2, 6]. Moreover, their identification in other pathological conditions and in healthy subjects is very rare (less than 0.5%) [7]. Furthermore, the identification of anti-dsDNA in SLE patients several years before disease onset suggests their involvement towards a clinically overt disease [8].

Several lines of evidence demonstrate the pathogenic role of anti-dsDNA antibodies. In particular, these autoantibodies have been associated with kidney involvement, as demonstrated by their deposition in several renal structures in SLE patients with active nephritis, that is, glomeruli, subendothelial and subepithelial spaces, mesangium, basement membrane, and tubules [9]. Moreover, by the interaction with toll-like receptor 9 (TLR 9), anti-dsDNA complexed with DNA could determine the activation of dendritic cells, with consequent B and T-cells activation and the release of proinflammatory cytokines [10]. Data from the literature demonstrate that the increase in anti-dsDNA serum levels could precede the relapse of disease, especially in terms of renal disease exacerbation [11, 12].

Despite the central role of these antibodies in the disease pathogenesis, a percentage of SLE patients, ranging from 2 to 30%, result negative for anti-dsDNA [7]. In the light of these considerations, the present study evaluated the clinical and laboratory features and therapeutical approach in a large monocentric SLE cohort, grouping patients according to their anti-dsDNA* status* and performing a comparison among the different subgroups.

## 2. Patients and Methods

We enrolled SLE patients referred to the Lupus Clinic of the Rheumatology Unit, Sapienza University of Rome (*Sapienza Lupus Cohort*). The diagnosis was performed according to the revised 1997 American College of Rheumatology (ACR) criteria [13].

Patients provided a written informed consent at the time of the first visit. The local ethical committee of “Policlinico Umberto I,” Rome, Italy, approved the study. At each visit, the patients underwent a complete physical examination. The clinical and laboratory data were collected in a standardized, computerized, and electronically filled form, including demographics, past medical history with date of diagnosis, comorbidities, and previous and concomitant treatments.

We assessed the disease activity by using the European Consensus Lupus Activity Measurement (ECLAM) [14], since this index does not include the measurement of anti-dsDNA antibodies.

### 2.1. Clinical Evaluation of SLE Patients

According to 1997 ACR revised criteria [13], we registered the presence of the following SLE manifestations:

- *Skin Involvement*. Malar rash (fixed erythema, flat or raised, over the malar eminences, tending to spare the nasolabial folds), discoid rash (erythematous raised patches with adherent keratotic scaling and follicular plugging; atrophic scarring may occur in older lesions), and photosensitivity (skin rash as a result of unusual reaction to sunlight, by patient history or physician observation).
- *Oral Ulcers*. Oral or nasopharyngeal ulceration observed by physician.
- *Serositis.* Pleuritis (convincing history of pleuritic pain or rubbing heard by a physician or evidence of pleural effusion) or pericarditis (documented by electrocardiogram or rub or evidence of pericardial effusion).
- *Kidney Involvement*. Persistent proteinuria >0.5 grams per day or > than 3+ if quantitation not performed or cellular casts (red cell, hemoglobin, granular, tubular, or mixed).
- *Neurologic Disorder*. Seizures (in the absence of offending drugs or known metabolic derangements, e.g., uremia, ketoacidosis, or electrolyte imbalance) or psychosis (in the absence of offending drugs or known metabolic derangements, e.g., uremia, ketoacidosis, or electrolyte imbalance).
- *Hematologic Disorder*. Hemolytic anemia with reticulocytosis or leukopenia <4.000/mm^3^ on ≥2 occasions or lymphopenia <1.500/mm^3^ on ≥2 occasions or thrombocytopenia <100.000/mm^3^ in the absence of offending drugs.

Clinical manifestations were cumulative and referred to the disease history.

### 2.2. Laboratory Evaluation

The study protocol included the determination of autoantibodies and the evaluation of C3 and C4 serum levels. Specifically, ANA has been determined by means of indirect immunofluorescence (IIF) on HEp-2 (titer ≥1 : 160 or ++ on a scale from + to ++++), anti-dsDNA with IIF on* Crithidia luciliae* (titer ≥1 : 10), ENA (including anti-Ro/SSA, anti-La/SSB, anti-Sm, and anti-RNP) by ELISA assay considering titers above the cut-off of the reference laboratory, anti-cardiolipin (anti-CL) (IgG/IgM isotype) by ELISA, in serum or plasma, at medium or high titers (e.g., >40 GPL or MPL or above the 99th percentile), anti-*β*2 Glycoprotein-I (anti-*β*2GPI) (IgG/IgM isotype) by ELISA, in serum (above the 99th percentile), and lupus anticoagulant (LA) according to the guidelines of the International Society on Thrombosis and Hemostasis (scientific subcommittee on lupus anticoagulant/phospholipid-dependent antibodies) [15]. Finally, C3 and C4 serum concentrations were studied by means of radial immunodiffusion.

According to the anti-dsDNA* status*, we identified three groups of patients:

- Anti-dsDNA +: SLE patients with persistent positivity.
- Anti-dsDNA ±: SLE patients with initial positivity and subsequent negativity during disease course.
- Anti-dsDNA −: SLE patients with persistent negativity.

We evaluated all the patients at the last visit in our Lupus Clinic. The antibodies* status* was assessed during the whole disease course; consequently, antibodies* status* follow-ups corresponded to the disease duration.

### 2.3. Statistical Evaluation

We used version 13.0 of the SPSS statistical package. Normally distributed variables were summarized using the mean ± standard deviation (SD) and nonnormally distributed variables were by the median and range. Percentages were used when appropriate. Mann-Whitney test was performed accordingly. Univariate comparisons between nominal variables were calculated using chi-square test or Fisher's test where appropriate. Two-tailed *P* values were reported; *P* values less than 0.05 were considered significant.

## 3. Results

In the present study, we evaluated 393 SLE patients [29M/364F (7.4%/92.6%); 386 (98.2%) Caucasian; mean age ± SD 44.8 ± 13.0 years; mean disease duration ± SD 152.4 ± 104.4 months]. Two hundred ninety-seven patients (75.6%) showed a persistent or previous positivity for anti-dsDNA. When grouping patients according to the anti-dsDNA* status*, 245 patients (62.3%) were anti-dsDNA +, 52 (13.3%) anti-dsDNA ±, and 96 (24.4%) anti-dsDNA −. Regarding anti-dsDNA ± subjects, anti-dsDNA antibodies became negative after a mean period from the diagnosis of 8.5 ± 8.3 years.

As reported in Table 1, no significant differences among the three groups of patients were identified concerning the sex distribution, the mean age, and the mean disease duration.

**Figure 1:**
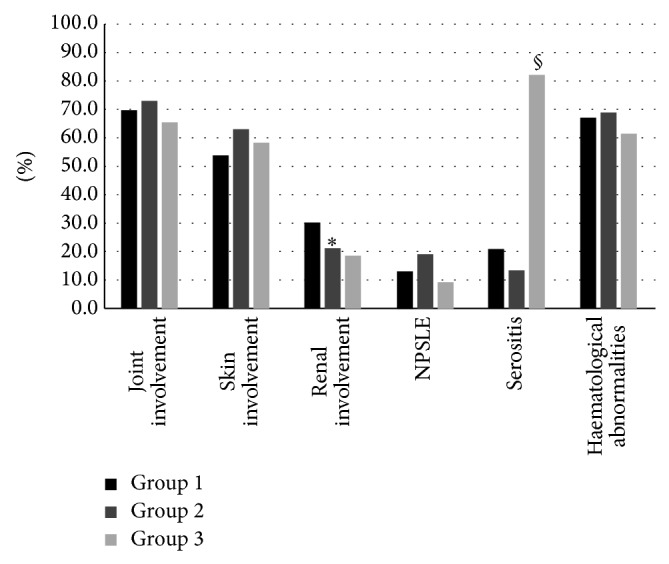
Clinical features of the 245 (62.3%) anti-dsDNA + (group 1), 52 (13.3%) anti-dsDNA ± (group 2), and 96 (24.4%) anti-dsDNA − (group 3) SLE patients. ^∗^ *P* = 0.001 group 1* versus* group 2 and group 1* versus* group 3; ^§^ *P* < 0.0001 group 3* versus* group 1 and group 3* versus* group 2.

**Figure 2:**
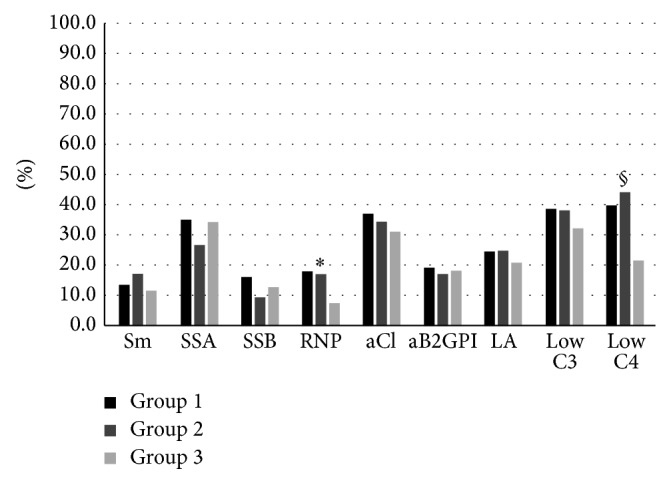
Immunological features distribution in the anti-dsDNA + (group 1), anti-dsDNA ± (group 2), and 96 (24.4%) anti-dsDNA − (group 3) SLE patients. ^∗^ *P* = 0.04 group 1* versus* group 3 and group 2* versus* group 3; ^§^ *P* = 0.005 group 1* versus* group 3 and group 2* versus* group 3.

**Figure 3:**
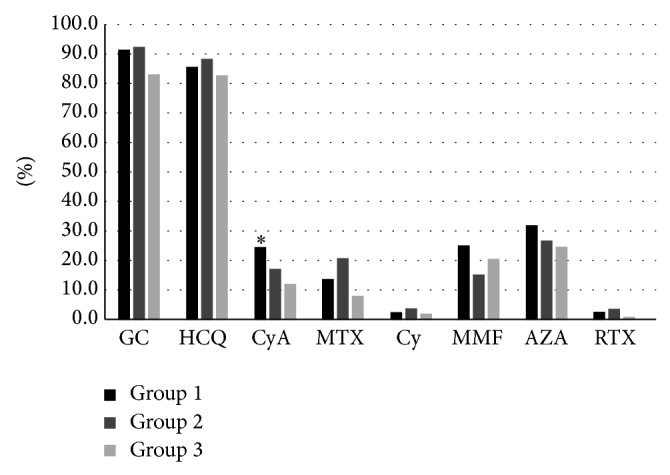
Therapies distribution of the 245 (62.3%) anti-dsDNA + (group 1), 52 (13.3%) anti-dsDNA ± (group 2), and 96 (24.4%) anti-dsDNA − (group 3) SLE patients. ^∗^ *P* = 0.01 group 1* versus* group 2 and group 1* versus* group 2.

We evaluated data concerning the distribution of the clinical features (Figure 1), laboratory parameters (Figure 2), and therapies (Figure 3) in the three groups of subjects.

The renal involvement was significantly more frequent in the anti-dsDNA + patients (73 patients, 30.2%) compared to anti-dsDNA ± (11 patients, 21.1%) and anti-dsDNA − (18 patients, 18.7%) (*P* = 0.001 for both comparisons, Figure 1). Conversely, serositis resulted significantly more frequent in the anti-dsDNA − (79 patients, 82.3%) compared to the anti-dsDNA + and anti-dsDNA ± (51 (20.8%) and 7 patients (13.4%), resp.; *P* < 0.0001, Figure 1).

Concerning the immunological abnormalities (Figure 2), the different autoantibodies showed a similar distribution in the three groups except for the anti-RNP which were significantly more frequent in the anti-dsDNA + and the anti-dsDNA ± groups [45 (18.2%) and 9 (17.3%) patients, resp.], compared with the anti-dsDNA − [7 patients (7.5%), *P* = 0.04 for both comparisons]. Similarly, the reduction of C4 serum levels resulted more frequent in the anti-dsDNA + and anti-dsDNA ± [98 (40.0%) and 24 (44.2%) patients, resp.] than in the anti-dsDNA – (21 (21.8%) patients; *P* = 0.005 for both comparisons, Figure 2).

In the anti-dsDNA +, we performed a comparison between patients with and without anti-RNP antibodies: patients with anti-RNP + showed more frequently skin manifestations compared with those of anti-RNP negative (70.0% versus 49.3%, *P* = 0.02). Moreover, the frequency of anti-Sm was higher in patients with anti-RNP compared with negative patients (57.5% versus 4.6%, *P* < 0.0001).

Finally, a similar therapeutical approach was applied in the three patients groups, with similar percentage of immunosuppressant drugs, except for cyclosporine A which was more frequently prescribed in the anti-dsDNA + patients (60 patients, 24.5%) compared to anti-dsDNA ± and anti-dsDNA − patients (9 (17.3%) and 12 (12.5%) patients, resp.; *P* = 0.01; Figure 3).

**Figure 4:**
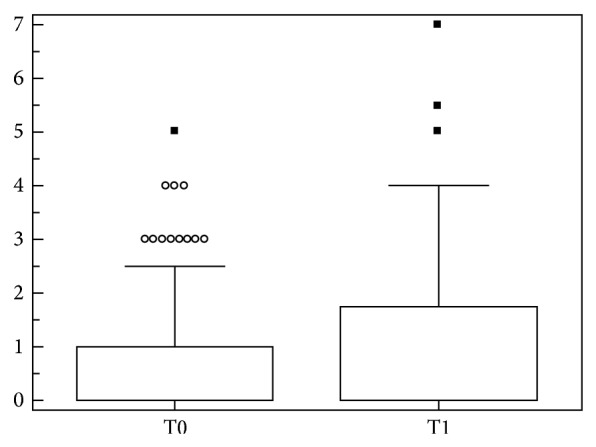
Mean ECLAM values before (T0) and after (T1) modification of anti-dsDNA* status*. Box and whiskers plot (median, quartiles, range, and possible extreme values).

Moreover, we focalized our attention on anti-dsDNA ± (SLE patients with initial positivity and subsequent negativity during disease course). In order to assess the disease activity changes, we evaluated the mean ECLAM values before (mean follow-up 8.5 ± 8.3 years) and after (mean follow-up 4.3 ± 2.1 years) anti-dsDNA modification. No significant differences were identified in the mean ECLAM values before and after the return to negative results (1.0 ± 1.3* versus *0.8 ± 0.9, *P* = 0.7; Figure 4). Moreover, the comparison of mean ECLAM values between anti-dsDNA ± and anti-dsDNA + patients did not show any significant difference (0.9 ± 1.05* versus *1.0 ± 0.9, *P* = 0.8).

This result was confirmed indirectly by the evaluation of treatment in this group of patients: 46.1% of the patients maintained the same treatment regardless of the anti-dsDNA modification. Nonetheless, 17.3% of the population who become anti-dsDNA negative required the introduction of a new immunosuppressant treatment.

## 4. Discussion

The results of the present study identified an association between the persistent or previous positivity for anti-dsDNA and specific clinical (kidney involvement) and immunological features (reduction of C4 serum levels, positivity for anti-RNP antibodies). Conversely, patients negative for anti-dsDNA seem to show a clinical picture characterized by a higher prevalence of serositis. Moreover, the patients experiencing the modification of anti-dsDNA* status* during disease course do not appear to represent a specific subgroup.

SLE is an autoimmune disease, potentially involving any organ/system, with a remitting-relapsing course [2, 16]. From a pathogenetic point of view, the production of several autoantibodies characterizes the disease. Among these, anti-dsDNA represent the hallmark of SLE patients having a diagnostic value given their strong specificity [6, 7]. Moreover, the presence of anti-dsDNA has been associated with a more severe disease pattern characterized by renal involvement, and a titer increase may predict a disease relapse [11, 12].

Despite these considerations about the role of anti-dsDNA, a growing interest is devoted to other antibodies detected in the serum of SLE patients, as evident by the classification criteria recently proposed by the Systemic Lupus International Collaborating Clinics [17, 18]. Indeed, this new classification is characterized by the modification of immunological items: autoantibodies other than anti-dsDNA, such as anti-Sm, LA, anti-CL, and anti-*β*2GPI, have been considered as a single criterion, determining the greater weight of these antibodies in the classification of SLE patients [18].

On the other hand, data from the literature suggest an association between different autoantibodies and specific clinical manifestations such as anti-dsDNA and lupus nephritis, anti-SSA/SSB and* sicca* symptoms, and anti-RNP and Raynaud's phenomenon [7]. To and Petri in 2005 identified different autoantibody clusters in a large cohort of SLE patients. The authors suggested that the Sm/RNP cluster represents the most benign subset, with more frequent skin involvement and less common renal and hematological manifestations. Conversely, the cluster anti-dsDNA/LA/anti-CL is characterized by neuropsychiatric manifestations and thrombotic events [19].

In the present cohort, we registered a frequency of anti-dsDNA greater than 70% that is similar to the data reported in the literature for other Caucasian SLE populations [7]. The persistent or previous positivity* status* for anti-dsDNA seems to identify a SLE subset characterized by the positivity for anti-RNP and the reduction of C4 serum levels. Moreover, the significantly higher frequency of renal involvement in persistently positive patients is in agreement with data from the epidemiological studies, thus confirming the pathogenetic role of anti-dsDNA in the kidney injury [9, 11, 20]. Furthermore, the association with low C4 serum levels confirms the link between anti-dsDNA and complement in SLE patients with renal manifestations [21]. Recent evidence suggests the influence of the complement receptors in the development of anti-dsDNA by participating in clearance of immune complexes and/or modulating B cells activation in response to antigen [22].

The association with a higher frequency of anti-RNP could be difficult to interpret. As known, patients affected by mixed connective tissue disease are frequently positive for these autoantibodies, with a frequency reaching 100% [7]. In SLE patients, the presence of anti-RNP ranges from 10 to 30% and is associated with specific manifestations, such as arthritis and Raynaud's phenomenon [7].

Moreover, according to data from the abovementioned analysis conducted by To and Petri, the Sm/RNP cluster seems to be the most benign subset, with less common renal involvement [19]. In our cohort, the presence of joint involvement and Raynaud's phenomenon is similar in the three groups of patients. These results could be explained by the different ethnicity in the cohorts evaluated. In the previous cohort, less than 60% of SLE patients evaluated were Caucasian; conversely, almost all SLE patients evaluated in the present analysis are Caucasian [19].

Moreover, we analyzed the group of SLE patients persistently negative for anti-dsDNA, identifying a significantly higher frequency of serositis compared to the anti-dsDNA positive patients. Data from the literature describe serositis as a frequent SLE manifestation, especially as pericarditis (8–48% of patients) and pleurisy (30–45%) [23]. In our analysis, the frequency of serositis in persistently anti-dsDNA negative patients resulted higher than 80%. Several authors have reported the association between serositis and positivity for anti-SSA/SSB antibodies, unconfirmed in our cohort [7, 24]. Certainly, a limit to consider is the relatively small number of patients in this group (96 subjects), determining caution in the interpretation of the results. In the light of the features of this group, a better analysis, by assessing larger population, could be very attractive in order to characterize these SLE patients and to modify some aspect strictly related to the positivity for anti-dsDNA. For example, biological drugs, such as belimumab, could be prescribed exclusively in patients with positivity for anti-dsDNA, considering active disease only in these patients.

A different genetic background could explain these differences. SLE is a multifactorial disease in which genetic and environmental factors interplay, determining disease development [2, 25, 26]. The genetic background could explain not only the disease susceptibility but also the autoantibodies production. The genome-wide association study, conducted by Chung and colleagues in 2011, demonstrated that many previously identified SLE-associated genes are more strongly associated with the production of anti-dsDNA than with disease susceptibility [27]. The authors demonstrated the association between polymorphisms (SNPs) located in the MHC, STAT4, IRF5, and ITGAM regions and the positivity for anti-dsDNA antibodies. Conversely, only SNPs in the MHC and IRF5 regions have been identified in negative patients [27]. These results suggested that some genetic variants could be considered “autoantibody propensity loci” rather than “SLE susceptibility loci” [27]. Finally, for the first time the present study analyzed a peculiar group of SLE patients, those with initial positivity and subsequent negativity of anti-dsDNA. Some points to consider were derived from the evaluation of this group. Firstly, the only clinical and laboratory difference identified in patients who become negative for anti-dsDNA compared to persistently positive patients was a lower frequency of renal involvement. Moreover, the modification of autoantibodies status was not associated with a change in disease activity, as demonstrated by the absence of significant difference in the mean ECLAM values before and after the status change. We have chosen to assess disease activity by ECLAM, because this index does not include the anti-dsDNA determination among the items evaluated, unlike other disease activity indices such as SLE Disease Activity (SLEDAI) [28]. This observation is reinforced by the evaluation of the therapeutical strategies adopted in this group of patients after the modifications of the anti-dsDNA status. Almost half of the patients maintained the same treatment and 17.3% required the introduction of a new immunosuppressant drug. Taken together these results suggest that the presence of anti-dsDNA is associated with a specific subset of disease with peculiar clinical and laboratory features, which do not change when anti-dsDNA become negative, maintaining similar aspect also in terms of disease activity. On the contrary, the persistently anti-dsDNA negative status seems to identify another subset of patients, with peculiar clinical features, in particular serositis.

## Figures and Tables

**Table 1:** Demographic characteristics of the SLE patients (*N* = 393) according to the anti-dsDNA *status*.

	Anti-dsDNA + N = 245	Anti-dsDNA ± *N* = 52	Anti-dsDNA − *N* = 96	P
Female – n (%)	230 (93.9)	47 (90.4)	87 (90.6)	**a**, **b**: P = 0.2; **c**: P = 0.5
Age (mean ± SD, years)	44.9 ± 13.6	43.8 ± 11.9	45.2 ± 12.2	**a**, **c**: *P* = 0.2; **b**: P = 0.8
Disease duration (mean ± SD, months)	12.6 ± 8.8	14.5 ± 9.5	11.9 ± 8.0	**a**: P = 0.4; **b**: P = 0.1; **c**: P = 0.3

**a**: anti-dsDNA + versus anti-dsDNA ±; **b**: anti-dsDNA ± versus anti-dsDNA −; **c**: anti-dsDNA + versus anti-dsDNA −.
